# Classification Challenges in Heterogeneous High Intellectual Potential Profiles and Autism without Intellectual Disability: When Similar Behaviors May Reflect Distinct Self–Other Mechanisms

**DOI:** 10.1192/j.eurpsy.2026.11109

**Published:** 2026-07-31

**Authors:** N. Lavenne, P. Planche, L. Vaivre-Douret

**Affiliations:** 1 Doctoral School 261-3CH, University of Paris Cité, Faculty of Societies and Humanities, Institute of Psychology; 2 INSERM UMR1018-CESP, Faculty of Medicine, University of Paris-Saclay, UVSQ, Villejuif, Research Team “Neurodevelopmental and Learning Disabilities, Necker-Enfants Malades Hospital, Paris; 3 INSERM UMR 1101 Laboratory of Medical Information Processing (LaTIM), University of Western Brittany; 4 Department of Child and Adolescent Psychiatry, Brest University Hospital; 5 Faculty of Medicine, University of Western Brittany; 6 CREAD Laboratory, EA 3875, University of Western Brittany, Brest; 7 Faculty of Health, Department of Medicine, University of Paris Cité; 8 Institut Universitaire de France (IUF), Chair of Neurodevelopmental Clinical Phenotyping; 9 AP-HP, Necker-Enfants Malades University Hospital; 10 IMAGINE Institute, Department of Endocrinology, Necker-Enfants Malades Hospital, Paris, France

## Abstract

**Introduction:**

High Intellectual Potential (HIP) and Autism Spectrum Conditions without intellectual disability (ASC-noID) often show overlapping traits (e.g., sensory hypersensitivity, social rigidity, pedantic language). With broadened autism criteria and growing recognition of neurodiversity, these similarities generate clinical uncertainty. Clinicians may struggle to determine whether behaviors reflect autism, giftedness, or both, raising the risk of misclassification, inappropriate interventions, and delayed support.

**Objectives:**

This work aims to clarify the apparent overlap between HIP and ASC-noID by examining the processes that give rise to clinical similarities. Specifically, we sought (i) to identify mechanisms distinguishing HIP and ASC-noID beyond surface resemblance, (ii) to explore how regulatory processes shape empathic functioning, and (iii) to discuss implications for clinical assessment and individualized support.

**Methods:**

We conducted a focused literature review and theoretical analysis of socio-cognitive and affective profiles across HIP, ASC-noID, and twice-exceptional (HIP+ASC) individuals. A neurophenomenological perspective was adopted, emphasizing empathy regulation and the Self–Other Distinction (SOD)—the capacity to resonate with others while maintaining a differentiated sense of self—as central to explaining similarities that often confound classification.

**Results:**

Behavioral overlaps appeared to stem from divergent mechanisms. In ASC-noID, social and communication atypicalities frequently stem from early neurodevelopmental alterations in bodily self-awareness, multisensory integration, and intersubjective attunement, producing persistent challenges with flexible social understanding; camouflaging (masking) may further obscure difficulties in clinical settings. By contrast, some HIP individuals—especially those with uneven cognitive profiles—may display autistic-like behaviors driven by anxiety, sensory overload, perfectionism, or compensatory overregulation, rather than by core social-cognitive differences. Theory of Mind tasks often fail to capture these distinctions. An SOD-gradient interpretation suggests that lower SOD flexibility may correspond to stronger ASC-noID symptom expression and reliance on camouflaging, whereas higher SOD flexibility in HIP supports perspective-taking but may involve costly overregulation and emotional fatigue.

**Conclusions:**

This analysis underscores the limits of categorical diagnostic approaches and supports a dimensional, regulation-focused perspective. Superficial resemblance between HIP and ASC-noID should not be taken at face value. Considering context, regulatory effort, compensatory strategies, and SOD-related dynamics can improve assessment, reduce misclassification, and guide tailored interventions.

**Disclosure of Interest:**

None Declared

